# A Mini Narrative Review on Human DNA Transfer Involving Dogs and Cats and Their Role in Forensic Investigation

**DOI:** 10.3390/genes17040423

**Published:** 2026-04-02

**Authors:** Carla Bini, Alessia Trasatti, Arianna Giorgetti, Sara Amurri, Giulia Fazio, Susi Pelotti

**Affiliations:** Section of Legal Medicine, Department of Medical and Surgical Sciences, University of Bologna, 40126 Bologna, Italy; carla.bini@unibo.it (C.B.); alessia.trasatti@studio.unibo.it (A.T.); sara.amurri4@unibo.it (S.A.); giulia.fazio2@unibo.it (G.F.)

**Keywords:** human DNA transfer, domestic animals, crime scene investigation

## Abstract

Background/Objectives: The potential role of domestic animals in DNA transfer, persistence, prevalence and recovery (TPPR) warrants careful consideration in forensic contexts. This mini narrative review aims to provide an updated overview of human DNA transfer involving household dogs and cats as vectors, to clarify their forensic relevance, and to identify key considerations for the design of future experimental research. Methods: A narrative review was conducted using multiple electronic databases as search engines without restriction related to the timing of publication. Results: Experimental evidence shows that dogs and cats readily acquire human DNA following even brief contact, acting as reservoirs for primary DNA transfer. Once acquired, human DNA can be redistributed via secondary transfer to a wide range of substrates, such as gloved hands, vehicle interiors, clothing, and surfaces. Moreover, multi-step and higher-order transfer events have been documented, highlighting the complexity of DNA transfer involving household animals. Conclusions: The sampling on pets may be included in certain scenarios and may contribute to building a Bayesian network together with the experimental data. To deal with uncertainty during probability assignment, more experimental data, especially addressing the main variables impacting DNA TPPR involving pets, should be generated and are highly needed to assist in activity level evaluation.

## 1. Introduction

In recent years, increasing attention has been directed toward the factors influencing DNA transfer, persistence, prevalence, and recovery (TPPR) in forensic contexts [1,2]. A persistent limitation lies in the availability of experimental data under conditions relevant to criminal cases even if an increasing number of publications dealing with these issues was observed [3].

As the prevalence of companion animals in domestic settings continues to increase, their potential role in human DNA transfer warrants careful consideration, especially in crime investigation where pets could be vectors of biological evidence.

The sustained and intimate contact between humans and their pets, as well as the interaction of companion animals with intruders and criminal offenders [4] creates favorable conditions for the deposition, accumulation, and persistence of human biological material on animals and on items with which they interact. From a forensic genetics’ perspective, these dynamics raise important considerations regarding DNA transfer mechanisms and evidence interpretation in forensic casework [5].

Recent studies have demonstrated that both dogs and cats can harbor detectable quantities of human DNA, predominantly originating from individuals within the household, although contributions from non-household members are also frequently observed [6]. Companion animals may act not only as passive carriers of human DNA, but also as intermediates facilitating its transfer between individuals, objects, and environments [7].

The aim of the present mini narrative review is to provide an updated overview of human DNA transfer involving household pets as vectors, particularly examining the experimental approaches employed in existing studies and evaluating the feasibility and extent of primary, secondary or higher-order transfer. By conducting a methodological synthesis of the published articles on the topic, and considering the multiple variables involved in DNA TPPR, we also aim to identify potential research gaps which may need further investigation, and to discuss the implications of the experimental results for activity level propositions.

Finally, by synthesizing current evidence, the present work aims to enhance understanding of the forensic relevance of companion animals in criminal investigation and to identify key considerations for the design of future experimental research in this emerging field.

## 2. Materials and Methods

A narrative review was conducted until the end of February 2026 using multiple electronic databases as search engines, particularly PubMed, Scopus, and Web of Science, including articles exploring the topic of DNA transfer using household pets. Search terms used were “forensic”, “DNA transfer” combined with “pet”, “dog”, “cat”, “domestic animal”. No restriction related to the timing of publication was applied. Only articles published in English were considered, while book chapters were not included.

References to the included articles were cross-checked for inclusion.

From the included articles, the following data were extracted: authors and year of publication, type of article, whether original article or short communication/conference proceedings, experimental design, technical characteristics, including sampling methods, DNA extraction, quantification and amplification, and main findings of the study. Experimental design and main findings of the studies were briefly summarized by the authors.

The screening of the databases was conducted by multiple reviewers, and disagreements were managed to uniform the terminology, deciding to adopt the authors’ one. No gray literature was included because it was missing.

## 3. Results

The present mini review identified a limited number *(n* = 6) of articles specifically addressing human DNA transfer involving pet and household animals, summarized in Table 1.

Among these, three studies focus on dogs, two on cats, and one included both. The emergence of the research topic of human DNA transfer involving domestic animals as vectors appears recent, since all articles were published from 2022 onwards [5].

## 4. Discussion

The recent concentration of publications on human DNA transfer involving domestic animals suggests an increasing awareness of the potential forensic relevance of primary and secondary DNA transfer involving domestic animals, particularly in indoor environments. On the other hand, the limited number of studies and their recent publication dates highlight the preliminary nature of this research field and underline the need for further investigation to better characterize the human DNA transfer via household pets, as recommended by the authors in the forensic context.

### 4.1. Experimental Design

The experimental designs described in the reviewed studies involved a sample size between 5 and 20 animals and were based on a common framework of either background (naturalistic) conditions or controlled or semi-controlled models simulating typical human–domestic animal interactions. In the latter, dogs and cats were involved in contact scenarios with owners, household members, external individuals, and selected surfaces or objects to assess human DNA transfer.

Sampling was primarily focused on fur-covered areas, with samples collected immediately after contact or at short post-contact intervals. The presence and origin of background human DNA on domestic cats was first assessed in a preliminary study published in 2022, involving naturalistic conditions of 15 households [5]. In this study, sampling was limited to the cat’s right side, with no further specification. In the following study of Monkman H et al., 2023, both the surface of the fur and the skin beneath it were sampled, demonstrating higher human DNA quantities from the former [8]. This may be explained by the fact that the fur of animals is more exposed to the environment and is therefore more likely to come into contact with humans and their surrounding surfaces, compared to the inner surface, which is protected by fur [9]. The top of the fur is thus recommended for sampling to recover human DNA.

Multiple sites of sampling were analyzed in four studies, two involving dogs, one cat and one both, with head and back providing the best human DNA recovery both for dogs [8] and cats [9]. These areas are thus suggested for recovery of human touch DNA on companion animals. Interestingly, although domestic animals also use to ingest and lick the fur on the stomach area, significantly less DNA was recovered on dogs from this area compared to other sampling areas on fur [8], so that the sampling from stomach area is not suggested for future studies on dogs.

The characteristics of the fur, e.g., length and porosity, the shedding abilities of the owners and animals as well as differences between indoor and outdoor pets, to the best of the authors knowledge, have not been extensively investigated in experimental studies yet. Fur length, classified as short, medium, or long, was mentioned in the 2022 study involving cats, but no significant difference in the amount of human DNA was found depending on this characteristic [5]. Similarly, the dog breeds and the fur length were specified for dogs in the most recent study, but these variables were not used for correlation analysis [7].

Factors that certainly influence the amount of DNA recovered, and should thus be considered in an experimental design, include washing time frames and human proximity and interactions [8], with particular reference to the nature of a pat, where limited force is applied as opposed to the force typically applied when a person uses an item [11].

Brief contacts with holders or patters or scratchers were mostly simulated in the experiments, while persistence of human DNA has not been thoroughly investigated yet [7].

Moreover, scenarios with variable connections between pet owner and offender, different environmental and dog transportation conditions are suggested, especially in relationship to animal kidnapping cases [7].

### 4.2. Technical Issues

Five out of six articles included in our review (83%) involved a double wet–dry swabbing method, although no experimental comparison of DNA recovery efficiency between different swab types has been conducted on domestic animals. The double-swab technique has been indicated as preferable for DNA recovery from complex surfaces [12], especially in the case of touch DNA [13].

For background samples collection, among our series, Oefelein R. et al. was the only study using cotton swabs for human DNA collection from pets back, ears/head and nose/mouth [6], and showed lower DNA recovery from cats (average 0.11 ng) compared to other studies (median 0.22 ng [5], 0.36–0.33 ng on back and head [9]). On the other hand, human DNA recovered from dogs (average 0.98 ng [6]) was higher than the median reported by Monkman et al. (median 0.06, from 0 to 1.26 ng [8]), making it complex to clearly establish a preferred sampling strategy, as expected for the influence of the variables in the experimental design.

A recent study on wildlife specimens compared the effectiveness of human DNA touch recovery methods, particularly cotton swabs (used as wet swabs followed by a second dry swab), flocked swabs and foam swabs (both used as a single swab, with one wetted side followed by the dry side), as well as minitapes, showing that foam swabs significantly yielded higher DNA recovery for antelope fur [14]. In the absence of pet-specific validation studies, one might also consider extrapolating the best recovery method from research on human hair. To the best of the authors’ knowledge, there is no consensus on the optimal approach for recovering exogenous human DNA from human hair [15]. A study showed that the moistened swab, compared to other cleaning methods, was the most effective way to obtain both the hair and exogenous DNA from a human hair stained with biological material [16]. Nevertheless, findings from studies on pet fur may provide useful methodological insights for humans, rather than the reverse [15].

### 4.3. Background Human DNA and Profiles

The first study on background DNA on companion animals involved cats. The presence of human DNA was assessed on the right side of the pet in 80% of samples, with an average amount of 0.22 ng (range from 0 to 1.32 ng) [5].

A slightly lower percentage of samples with detectable quantities of human DNA (70.8%) was reported in the study of Monkman H. et al. on dogs [8]. Average amounts were lower, with a median of 0.06 ng, although this might be due to the multiple different sites sampled on dogs, including the stomach. Indeed, also when sampling multiple areas of cats, 69% of background samples provided detectable human DNA [9], highlighting the influence of anatomical sites on DNA deposition and recovery. When comparing background DNA on cats and dogs, Oefelein R. et al. found higher amounts on dogs (0.98 ng vs. 0.11 ng) and higher percentage of samples suitable for amplification (80% vs. 53%), and this was attributed to the available surfaces for sampling or different behavior of pets [6].

Nevertheless, washing the fur of the animal, as well as the characteristics of the shampoo and fur, could impact the presence and persistence of human DNA on household pets, similarly to hand cleaning that impacts the recovery of touch DNA from humans [17]. However, in the study by Monkman H. et al., the washing time frames, considered to interpret the results of DNA amount on dogs, did not impact the DNA recovery [8].

Variability in recovered DNA among different pets might additionally be related to different interactions between the owner and the pet, which also vary by the size of dogs, with smaller dogs having usually more intense contacts [8].

Other factors apparently did not impact the amount of human DNA recovered from pets, including the number of house occupants and amount of time spent with the pet, although the limited small dataset of the studies does not allow us to draw firm conclusions [8]. In most of the studies, persons from the household were detected in the majority of interpretable profiles, both on dogs [8] and cats, ranging in the latter from 66.7% to 79% [5,9], and up to 16 of 19 samples (84.2%) considering both pets [6].

In the first study by Monkman H. et al., although there was no significant difference between the amount of DNA present on the cat and the time since the cat was last contacted, the last person to touch the cat was mostly detected as the major or single source contributor [5]. Nevertheless, as suggested by the presence of a different number of contributors in two cats living in the same household, direct contact might not be the most impacting factor on human DNA presence. Other factors including the shedder status and the behavior of the cat, could be relevant for human DNA presence [5]. Again, in another study involving cats, the last person to have contact with the cat was detected in 61% of the samples [9].

Contrarily, considering dogs, the last person to contact the pet, as emerged from a questionnaire compiled by the carer of the dog, was only detected in 35% of samples, and higher DNA recovery was yielded from areas that were not the most recently contacted [8].

The observed dissimilarities between cats and dogs may be explained by differences between the typical cat–human and dog–human interaction: cats generally show limited active physical interaction with their owners, usually choose specific locations to rest and a preferred person to spend time with. In most cases, this preferred owner was also the last person to have contact with the animal prior to sampling, which could explain the greater accumulation of that individual’s DNA. Contrarily, dogs are closer and interact more with all their owners [9,18].

It is interesting to note that, despite reference profiles being obtained from the owner as well as from the other residents of the household in all studies, DNA from unknown sources was commonly observed in 47% of samples with five or more alleles in one study on cats [9], in 44.4% of interpretable profiles on cats in another study [5], and in 39.2% of interpretable profiles on dogs [8]. This was additionally confirmed in the simulated dog-theft experiment, where 70% of samples provided unknown DNA, with a total of 17 unknown donors detected across 14 samples out of 20, as already well-known from touch DNA and DNA transfer studies [7]. The high prevalence of unknown profiles could arise from the close interaction with the environment and with potentially contaminated surfaces usually contacted by pets, such as the floor [8]. Future research should investigate the role of realistic environmental factors, to minimize the gap between experimental and real-world scenarios relevant for forensic purposes.

### 4.4. Primary Transfer to Animals

**Figure 1:**
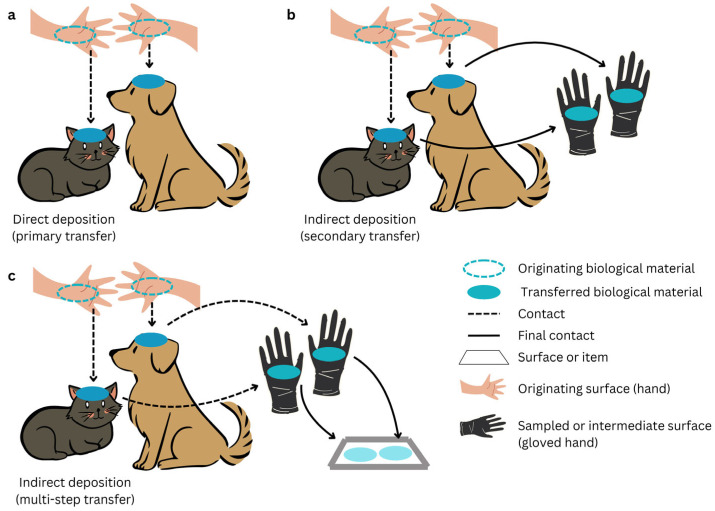
(**a**) An example of direct deposition or primary transfer. (**b**) An example of indirect deposition or secondary transfer. (**c**) An example of indirect deposition with multi-step transfer.

The studies included in the present mini review demonstrated that human DNA transfer could occur to dogs [7,8] and cats [9], even after a single contact, especially if it consists of scratching/patting the animal. An example of primary transfer or direct transfer is shown in Figure 1.

After a single scratch on the necks of dogs, median amounts of human DNA transferred were 0.09 ng, with a maximum of 0.4 ng, and 70.8% of samples showing human detectable DNA. In this case, 50% profiles matched the individual patting the dog [8].

A short one-time patting on cats, chosen to simulate a possible contact between an offender and the animal in a potential crime scene, did not always result in the detection of the patter’s DNA. Although quantifiable DNA was recovered from 75% of cats after contact with bare hands, the transfer from the patter to the cat was only detected in 25% of samples [9].

Higher percentages of transfer rate were observed in another recent experimental study, where holders were asked to pick up dogs and put them in a car, simulating a “dog—napping” scenario: the profile of the holder was identified in 40% of samples on dog [7]. The transfer of holders’ DNA occurred with contact of approximately 2 min, suggesting that the duration of the contact, as expected, influences the human TPPR [7]. Another possible influencing factor was hypothesized as the area of contact, since larger dogs had to be “secured” by the holder for transportation, and these dogs corresponded to the ones carried for longer times [7].

In another study, Monkman H. et al. [10] investigated the persistence and transfer of human DNA following brief human–dog interactions. After a five-minute pat-and-play interaction, DNA from the visitors or their housemate was recovered from the dog’s head, back, or sides in 50% of samples (10/20) one hour after contact. Visitor-associated DNA was never the major contributor, appearing only as a minor component in 35% of samples (7/20). Notably, DNA from a visitor’s housemate, who had no direct contact with the dog, was detected in 15% of samples (3/20), all of which originated from the same animal [10].

### 4.5. Secondary Transfer from Animals to Other Surfaces

A figure with an example of indirect or secondary transfer is shown in Figure 1. Detectable human DNA was transferred via dogs, used as vectors, to gloved hands scratching the animal on 95% of cases in the study of Monkman H. et al. DNA recovery on gloves ranged from 0.0 to 2.0 ng, with a median of 0.09 ng. Generated profiles mostly reflected the mixture of individuals found on the dogs’ fur [8].

In a study on cats, detectable and quantifiable DNA was found in 80% of samples collected from the gloves after contacting, with a recovery ranging between 0.0 and 0.72 ng. Even if not statistically significant, it emerged that cats with a higher level of background DNA were able to transfer higher amounts of DNA to the gloves. In most cases, the DNA belonged to the cat’s owner, consistent with cats’ tendency to prefer a specific owner and spend more time with them [9].

On the contrary, a maximum DNA amount of 0.4 ng was obtained from plastic sheets simulating the floor upon which dogs walked. This lower DNA amount might reflect DNA loss while walking, the small areas of paws contacting the floor, the background DNA on the plastic sheets simulating the floor or an area-related lower DNA persistence compared to other dogs’ surfaces, e.g., in the hypothesis of greater washing and licking of the paws by the dog, or extraction inhibition due to the dirt [8].

The secondary transfer on another plastic surface as plastic card, rubbed against the back, ears/head and nose/mouth of cats and dogs in the study by Oefelein R. et al. [6], showed very low DNA amounts (average 0.01 ng) and no profiles suitable for comparisons. The authors hypothesized that this result was due to use of a smooth surface, while other rough surfaces might be investigated.

In the “dog—napping” simulation, DNA of both the dog owners (35% detection rate) and of the dog holders (13% detection rate) was recovered from the surfaces of the car in which the dog was placed after the dog occupation, most likely indicating indirect transfer via the dogs [7]. The transfer of dog owners’ DNA was considered rather high compared to previous studies exploring indirect human DNA transfer under uncontrolled conditions [19,20]. The higher DNA transfer could be related to the higher shedder status of the participants even if not assessed in the study or to differences in activities involved, as reported by authors [7]. It is well known that the shedder status is one of the most important variables impacting the quantity of DNA deposited, with several papers demonstrating differences between good and bad shedder. The profile of good shedder has been found even on objects they never handled [2].

In the same experiment, indirect transfer from the dog owner to the shirt of the dog holders also occurred, with dogs acting as vectors, with a detection in 10% of the samples. Interestingly, one of the dog owners’ DNA was detected on dog, on the car where the dog was placed after pick up, and on the shirt of the dog holder, suggesting multi-step DNA transfer, considering that the study was performed under controlled conditions: the owner had not previously contacted the car or the holder on the t-shirt [7].

The study of Monkman H. et al. [10] investigating the impact of a five-minute pat-and-play interaction between humans and dogs demonstrated that dog owner DNA was transferred from the animal to the visitor, and subsequently to the visitor’s secondary touched surfaces in 33% of samples from the car and in 30% from kettle and mug touched by visitors at home. However, analysis of car surfaces contacted immediately after the visit revealed visitor’s DNA profile on all five car items as the major or predominant contributor and on the kettle and mug in 60% of the samples.

Data generated with similar experimental designs involving transfer events, as in the simulated “dog—napping” of Monkman H. et al. [7], were tested in only one study with a Bayesian network, but appeared to be informative for the DNA activity level evaluation.

Actually, nowadays the activity level evaluation represents a significant challenge for forensic genetics, which has shifted attention from the answer to the ‘Who?’ question to the ‘How?’ one. The application of Bayesian networks to inference problems involving the results of DNA analyses provides the likelihood of DNA findings given competing propositions of interest with respect to the activities aware of variables impacting DNA TPPR [2]. Improving the knowledge on TPPR through investigation and experimental data presentation may assist evaluation of evidence under propositions considering DNA transfer mechanism.

## 5. Conclusions and Future Perspectives

This review summarizes current evidence supporting the forensic relevance of domestic pets in primary and secondary human DNA transfer. Despite most studies involving a limited number of replicates, they provided data from various experimental designs simulating forensic scenarios and foundational methodologies for larger cohort studies.

The studies showed that household animals create multiple opportunities for direct but also multi-step indirect transfer of human biological material, with the need to differentiate between criminal activity-related transfer and innocent contacts. Overall, the complex dynamic of DNA transfer shows potential implications for the interpretation of forensic evidence in crime scenes. Actually, evaluation of a DNA profile from human biological traces requires us to determine the diagnostic value of findings given activity level propositions [21] that should consider the mechanisms of DNA TPPR able to impact the findings.

Recently, the novel term of contextual sampling was introduced to indicate the targeted collection of additional samples from the surroundings of crime-related items to assign probabilities on prevalence and background based on case-relevant data [21]. The sampling on pets may be included in certain scenarios within the contextual sampling and may contribute to some nodes of a Bayesian network, together with the experimental data informing additional nodes.

Notably, only one study within the reviewed literature implemented a Bayesian network approach to model transfer dynamics at the activity level, illustrating the potential of a probabilistic framework to account for competing propositions. Building on this approach, to deal with uncertainty during probability assignment, more experimental data should be generated and are highly needed to assist in activity level evaluation. Actually, variables can be combined in a Bayesian network to evaluate evidence with regard to the relevant activities at stake [22]. Further studies should follow The ReAct (Recovery, Activity) project, an ENFSI (European Network of Forensic Science Institutes) initiative with the primary purpose to design experiments simulating typical casework circumstances; collect data and to implement Bayesian networks to assess the value (i.e., likelihood ratio) of DNA results given activity level propositions [23]. As highlighted by the present mini review, future research should explore the main variables impacting DNA TPPR when pets are involved, as, among others, the transfer of primary touch DNA after different time periods, the awareness of general levels of background DNA [2] and also consider factors such as sampling method, prevalence and persistence of DNA, the influence of behavior of pets, environmental contamination, shedding status of the participants, and human–animal interactions in pet-involved crimes.

## Figures and Tables

**Table 1:** Summary of the studies reviewed, including authors, year of publication and type of article (Ref.), experimental design, technical procedures and main findings.

Ref.	Experimental Design	Technical Procedures	Main Findings
Monkman H. et al. 2022 [5] Short communication	A total of 20 cats from 15 households - **Samples on cats**: from the right side. - Reference samples from every person in household. Questionnaires on cats’ behaviors, living environment and recent human contacts.	Double swabbing technique. Extraction: DNA IQ System (Promega, Madison, WI, USA). Quantification: Quantifiler^®^ Trio system (Life Technologies, Carlsbad, CA, USA). DNA Amplification: PowerPlex^®^ 21 system (Promega, Madison, WI, USA).	- **Samples on cats**: 80% of samples showed detectable human DNA (average 0.22 ng). Interpretable profile in 70% of samples. - In 66.7% of interpretable profiles, one person from the household was detected. The last person to contact the cat was detected as the major or single source contributor. - DNA from unknowns was observed in 8/18 of interpretable profiles.
Monkman H. et al. 2023 [8] Original article	A total of 20 dogs, 9 samples per dog: - **Indirect transfer**: gloves after scratching and patting the dog and plastic floor after pet walking. - **Samples on dogs**: chest, head, back, left side under the fur and right-side surface of the fur, stomach. - **Direct transfer**: sample from dog neck after scratching with bare hand. Questionnaire to the primary carer of the dog regarding living conditions, interactions, and grooming. Reference samples: dog’s owners and residents of the household.	Wet–dry swabbing technique using viscose swabs. Extraction: DNA IQ System (Promega, Madison, WI, USA). Quantification: Quantifiler^®^ Trio. DNA system (Life Technologies, Carlsbad, CA, USA). DNA Amplification: PowerPlex^®^ 21 System (Promega, Madison, WI, USA).	- **Indirect transfer**: human detectable DNA on 19/20 occasions of secondary transfer to gloves (median 0.09 ng) and 9/20 to simulated floor (median 0.0 ng). - **Samples on dogs**: human detectable DNA in 70.8% (median 0.06 ng). Best recovery from the surface of the fur, head and back than under the fur. - Of 120 samples, in 61.7% the MNC was assigned (49 samples with two persons mixture, 7 samples, three persons mixture and 18 single source). - Of 120 profiles, 39.2% showed human DNA from unknown sources. - **Direct transfer** (from bare hands patting the dogs): occurred in 50% of cases.
Monkman H. et al. 2025 [9] Original article	A total of 20 cats, 6 samples per cat: - **Background samples**: the top of the fur cat’s head, back and right side, left side skin base of the fur. - **Secondary transfer**: palm and fingers of glove after scratching and patting the cat. - **Direct transfer**: cat’s chest and neck after scratching with bare dominant hand. Questionnaire to the carer of the cat regarding living conditions, habits, interactions and grooming.	Wet–dry double swabbing technique using viscose swabs. Extraction: DNA IQ System (Promega, Madison, WI, USA). Quantification: Quantifiler^®^ Trio DNA (Applied Biosystems, Waltham, MA, USA). DNA Amplification: PowerPlex^®^ 21 System (Promega, Madison, WI, USA).	- **Background samples**: detectable DNA from 69% of samples. Highest DNA yields from back fur (mean 0.36 ng), head fur (0.33 ng), right fur (0.22 ng), and skin at the base of the fur (0.06 ng). - In total, 29% did not produce a profile; 54% generated a profile with more than 4 alleles, of which 79% showed the owner’s profile. - DNA from an unknown source was detected in 47% of samples with more than 4 alleles. - **Secondary transfer** (from the cat to gloves) occurred in 80% of trials, yielding quantifiable DNA in 16 of 20 glove samples (mean 0.18 ng). DNA typing showed the owner profile in 12/14 of samples with more than 4 alleles. - **Direct transfer** (following brief patting with bare hands): 75% samples showed quantifiable DNA with a mean recovered amount of 0.11 ng. Donor DNA was transferred to the cat in 25% of the 20 samples collected.
Monkman H. et al. 2025 [10] Original article	A total of 5 dogs, visitors drove to the dog’s household, interacted for 5 min with the dog, patting and playing, returned to the car and drove home, where they were instructed to make tea. - DNA samples collected from the **first 5 surfaces** contacted **in the car** by visitors prior to home return. - DNA samples from the **first 3 items** touched by visitors on arrival at home. - DNA samples collected from the **kettle, mug** and **visitor’s hands** at home. - DNA samples collected from two areas where the dogs spent time post-interaction. - **Samples on dogs**: left side of the body, right side of the body, top of the head, top of the back. Questionnaire to the carer of the dog regarding the visitor’s living conditions, number of housemates and frequency of visitors.	Wet–dry double swabbing technique using viscose swabs. Extraction: DNA IQ System (Promega, Madison, WI, USA). Quantification: Quantifiler^®^ Trio DNA (Applied Biosystems, Waltham, MA, USA). DNA Amplification: PowerPlex^®^ 21 System (Promega, Madison, WI, USA).	- From the **first 5 surfaces** contacted **in the car** by visitors, the average DNA quantity was 4.25 ng. A DNA profile was generated from all surfaces. The dog owner’s DNA was detected in 33% of samples. - From the **first 3 items** touched by visitors at home, the average DNA recovered was 3.54 ng. 93% of samples generated a DNA profile. The dog owner was excluded from all of these samples. - From the **kettle and mug**, average DNA recovered was 1.37 and 2.70 ng, respectively. The dog owner profile was detected in 30% of these samples. - From the **visitor’s hands**, the average DNA recovery was 5.4 ng from dominant hand and 4.1 ng from a non-dominant hand. The dog owner profile was detected in 80% of these samples. - **Samples on dogs**: From the two areas where the dogs spent time post-interaction, average recovered DNA was 2.3 ng not showing the visitor’s profile. From the dog’s head, back or sides, quantifiable DNA was recovered in all samples (average 1.4 ng) and in 50% of samples the visitor’s or visitor’s related person was transferred.
Oefelein R. et al. 2025 [6] Short communication	A total of 10 domestic pets (5 canines and 5 felines) - **Background samples:** back, ears/head, nose/mouth. - **Secondary transfer**: 3 samples on a smooth plastic card rubbed on the animal’s same areas. LR calculation following propositions: (H1): Primary Pet Owner + N-1 Unknowns/ (H2): N Unknowns.	Single swabbing—cotton swab for pets. Double swabbing—wet and dry cotton swab for card samples. Extraction: Maxwell™ FSC DNA IQ™ Casework Kit with Promega Casework Extraction Kit (Promega, Madison, WI, USA). Quantification: PowerQuant^®^ System (Promega, Madison, WI USA). DNA Amplification: Powerplex Fusion 6C system (Promega, Madison, WI USA).	- **Background samples:** canines presented higher levels of background DNA (average 0.98 ng) than cats (average 0.11 ng). - In total, 53% of background feline samples and 80% of background canine samples were suitable for amplification. - In total, 31% of background samples were suitable for comparison supporting H 1 in 16/19 samples and H2 in 3/19. - **Secondary transfer**: human DNA was detected with an average DNA of 0.01 ng. - In total, 13% of secondary transfer feline samples and none of canine were suitable for amplification. - No sample produced DNA profiles suitable for comparison.
Monkman H. et al. 2026 [7] Original article	A total of 5 dogs, 5 cars, 1 holder, different shirt worn per experiment. Simulated dog-theft involving brief lifting and transporting by a handler, placing in an unfamiliar car (20 min), and return home by lifting and holding - **Background samples from cars** not associated with the dogs. - **Samples on dogs**: 1 h after simulated theft, 4 samples per dog (head, neck, sides). - **Samples from car** 4 h **after the simulated theft.** - **Samples of the holder’s t-shirt**: left, right arms, abdomen, chest worn by the holder the day after (*n* = 20). - DNA reference samples from the dog owners, car owners, car users and the holder. Questionnaires to owners on number of owners, preferred locations of the dog in house, regular activities. Bayesian network built.	Wet–dry double swabbing technique using viscose swabs and sterile water. Extraction: DNA IQ System (Promega, Madison, WI, USA). Quantification: Quantifiler^®^ Trio DNA (Applied Biosystems, USA) Quantifiler Kit on a 3500xL Genetic Analyser, GeneMapper™ ID-X Software v1.6Amplification: PowerPlex^®^ 21 System (Promega, Madison, WI, USA).	- **Background samples from the car**: in 9 samples DNA in quantity range 0.12–3.420 ng. - **Samples on dogs**: average DNA 0.79 ng, the least (av 0.36) from the left side. DNA profile from the owner (s) 85% of samples. DNA from the holder in 40%. Unknown DNA in 70% of the samples. - **Samples from car** 4 h **after the simulated theft:** DNA recovery on 18/19 samples of car (average quantity 1.81 ng). DNA of car owners in 47% of samples. DNA of dog owners in 35% of samples. DNA of the holder in 35% of samples and in 13% excluding the car belonging to the holder. - **Samples of the holder’s t-shirt:** DNA present in all the 20 samples (average 1.51 ng). DNA of car owners in 5% of samples (1 of 20) as minor contributor. DNA of dog owners in 10% of samples as minor contributor. The holder was detected in 85% of samples as sole or major contributor. At least one unknown contributor was detected in 75% of the samples as single source or minor contributor. Bayesian Network analysis demonstrated that dog-derived DNA can be useful for activity level propositions.

## Data Availability

No new data were created or analyzed in this study.
